# Chibby forms a homodimer through a heptad repeat of leucine residues in its C-terminal coiled-coil motif

**DOI:** 10.1186/1471-2199-10-41

**Published:** 2009-05-12

**Authors:** Adaobi Mofunanya, Feng-Qian Li, Jen-Chih Hsieh, Ken-Ichi Takemaru

**Affiliations:** 1 Department of Pharmacological Sciences, State University of New York at Stony Brook, Stony Brook, New York 11794, USA; 2 Graduate Program in Genetics, State University of New York at Stony Brook, Stony Brook, New York 11794, USA; 3 Department of Biochemistry and Cell Biology, Center for Developmental Genetics, State University of New York at Stony Brook, Stony Brook, New York 11794, USA; 4 Aderans Research Institute, 3401 Market Street, Philadelphia, Pennsylvania 19104, USA

## Abstract

**Background:**

The Wnt/β-catenin signaling pathway plays crucial roles in embryonic development and in maintenance of organs and tissues in adults. Chibby (Cby) is an evolutionarily conserved molecule that physically interacts with the key downstream coactivator β-catenin and represses its transcriptional activation potential. Although Cby harbors a predicted coiled-coil motif in the C-terminal region, its molecular nature and functional importance remain largely unexplored.

**Results:**

Here we report that Cby forms a stable complex with itself. Alanine substitutions of two or more of four critical leucine residues within the C-terminal heptad repeats completely eliminate the Cby-Cby interaction. The Cby oligomer predominantly exists as a homodimer. Furthermore, we found that dimerization-deficient Cby mutants still retain the ability to bind to β-catenin and to repress β-catenin-dependent gene activation. More importantly, Cby homodimerization is required for its efficient interaction with the nuclear import receptor importin-α and subsequent nuclear translocation.

**Conclusion:**

Our comprehensive mutational analysis of the Cby coiled-coil domain reveals that the four heptad leucine residues play an essential role in mediating Cby homodimerization. Although monomeric Cby is sufficient to bind to β-catenin and block β-catenin-mediated transcriptional activation, homodimer formation of Cby is indispensable for its efficient nuclear import.

## Background

Intracellular signaling activated by the Wnt family of secreted cysteine-rich glycoproteins is crucial for embryonic development, stem cell self-renewal and adult homeostasis [1-3]. More recently, dysregulation of Wnt signaling has been linked to a range of human diseases, especially cancer [4-6]. For instance, canonical Wnt/β-catenin signaling is aberrantly activated in greater than 70% of colorectal cancers, promoting cancer cell proliferation, survival and migration [7,8]. Accordingly, the Wnt/β-catenin pathway has gained recognition as an enticing molecular target for cancer therapeutics [9,10]. In this signaling cascade, β-catenin plays a pivotal role as a transcriptional coactivator [11,12]. In the absence of a Wnt ligand, cytoplasmic β-catenin becomes phosphorylated by casein kinase 1 (CK1) and glycogen synthase kinase 3 (GSK3) in a complex containing the tumor suppressors Axin and Adenomatous polyposis coli (APC), and is targeted for ubiquitin-mediated proteasomal degradation [13,14]. Wnt binding to the seven transmembrane Frizzled (Fz) receptors and the low-density lipoprotein receptor-related protein (LRP) co-receptors, LRP5 and LRP6, triggers recruitment of Axin to the plasma membrane, resulting in inhibition of β-catenin phosphorylation and degradation [15,16]. As a consequence, β-catenin accumulates in the cytoplasm and then translocates into the nucleus where it forms a complex with the T-cell factor/lymphoid enhancer factor (Tcf/Lef) family of transcription factors, leading to activation of target genes [17,18].

We previously reported a β-catenin antagonist Chibby (Cby) [19]. The human Cby protein is composed of 126 amino acids, and is highly conserved throughout evolution. Cby physically interacts with the C-terminal activation domain of β-catenin. Our recent crystal structural studies for a full-length β-catenin suggest that Cby binds to the Helix C located at the C-terminal end of the central Armadillo repeat region of β-catenin [20]. Cby functions as a repressor of β-catenin by competing with Tcf/Lef factors for β-catenin binding. Reduction of Cby protein levels in *Drosophila melanogaster *embryos by RNA interference (RNAi) results in hyperactivation of this pathway [19,21,22], underscoring the biological importance of Cby's function. Using *in vitro *cell culture models, we demonstrated that Cby facilitates adipocyte and cardiomyocyte differentiation of pluripotent stem cells through inhibition of β-catenin signaling [23,24]. More recently, we isolated 14-3-3 adaptor proteins as novel Cby-binding partners [25,26]. Upon phosphorylation of Cby serine 20 by Akt kinase, Cby and 14-3-3 form a stable trimolecular complex with β-catenin, and cooperate to promote cytoplasmic localization of β-catenin, leading to down-regulation of β-catenin-mediated transcriptional activation. Therefore, we proposed a new model in which inhibition of β-catenin signaling by Cby involves at least two distinct molecular mechanisms, i.e. competing with Tcf/Lef transcription factors for binding to β-catenin in the nucleus [19], and facilitating nuclear export of β-catenin via interaction with 14-3-3, following phosphorylation of Cby serine 20 by Akt [25].

Through comparison of Cby protein sequences across species, we noted that Cby contains a conserved putative coiled-coil motif in its C-terminal region [19]. In a later study, Hidaka *et al. *performed a yeast two-hybrid screen using full-length human Cby as bait, and pulled out Cby itself [27]. Their subsequent deletion analysis indicated that the C-terminal region of human Cby protein encompassing the coiled-coil domain (amino acids 60–112) is prerequisite for its self-association. However, the nature of Cby homooligomers and specific amino acid residues responsible for Cby self-assembly has remained to be elucidated.

In the present study, we report that Cby forms a stable homodimer via its C-terminal leucine zipper coiled-coil domain. Using a variety of Cby point mutants generated by site-directed mutagenesis, we found that four leucine residues in the C-terminal heptad-repeat region are responsible for Cby homodimerization. We also show that Cby mutants defective in homodimer formation are capable of both binding to β-catenin and antagonizing its signaling activity. More importantly, we provide evidence that Cby homodimerization is necessary for its efficient binding to the nuclear import receptor importin-α and subsequent nuclear import.

## Results

### Cby forms a stable oligomer

**Figure 1:**
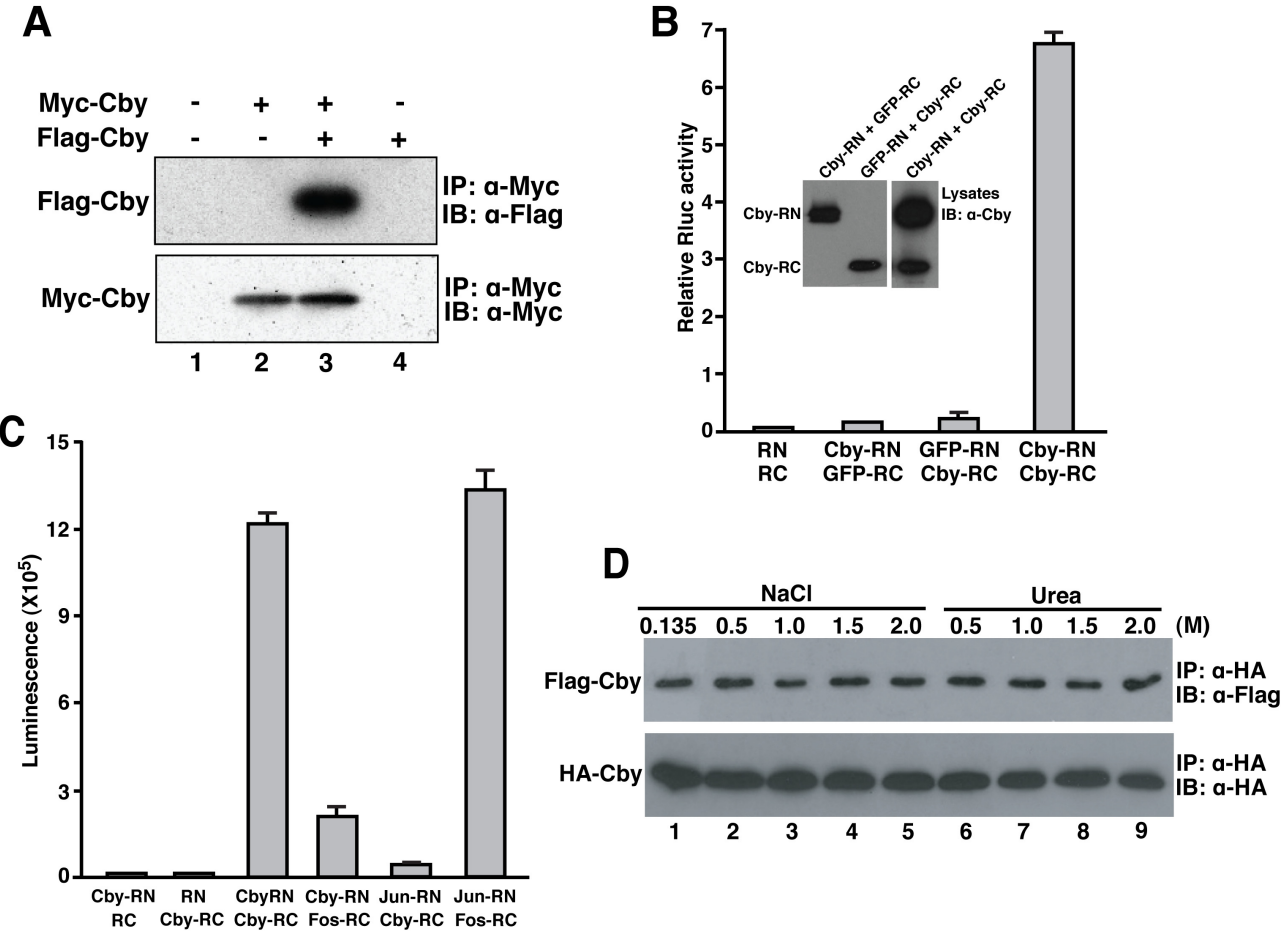
**Cby forms a stable complex with itself**. **(A) **Coimmunoprecipitation of Myc-Cby and Flag-Cby. **(B) **Cby self-interaction was detected by split synthetic Renilla luciferase (hRluc) protein-fragment-assisted complementation. Cby or negative control GFP was fused in-frame to the N-terminal portion (RN) and C-terminal portion (RC) of hRluc. These expression plasmids (400 ng each) were transfected into HEK293T cells as indicated, and Renilla luciferase (Rluc) activities were measured 24 hr post-transfection. A firefly luciferase plasmid (5 ng) was co-transfected to normalize transfection efficiency. Transfections were carried out in triplicate and the means ± SD are shown.**(C) **Cby-Cby interactions were detectable *in vivo *in real time using cell-permeable ViviRen live cell substrate. Cby-RN, Cby-RC, Fos-RC and Jun-RN were transiently expressed in HEK293T cells as shown, and ViviRen was added to the tissue culture media 24 hr post-transfection for luminescence measurements. Transfections were carried out in triplicate and the means ± SD are shown.**(D) **Cby complex is highly stable. The immunoprecipitates were washed three times with wash buffer containing 0.135, 0.5, 1.0, 1.5 or 2.0 M NaCl, or 0.5, 1.0, 1.5 or 2.0 M urea as shown. The experiments with urea were performed in the presence of 0.135 M NaCl.

Cby has been shown to interact with itself using the conventional yeast two-hybrid system [27]. In order to confirm and further extend this observation, we examined whether Cby forms a complex with itself in mammalian cultured cells by coimmunoprecipitation experiments (Figure 1A). Cell lysates from human embryonic kidney (HEK) 293T cells co-transfected with Myc- and Flag-tagged Cby expression constructs were immunoprecipitated with anti-Myc antibody and analyzed by Western blotting using anti-Flag antibody. A specific interaction was reproducibly observed between the Cby proteins (lane 3).

Next, split synthetic Renilla luciferase (hRluc) assays [28] were employed as an independent means to verify the Cby-Cby interaction. To this end, Cby or negative control GFP was fused in-frame to the N-terminal portion of hRluc (Cby-RN or GFP-RN) or to the C-terminal portion of hRluc (Cby-RC or GFP-RC). Physical interactions between fusion proteins would bring the N-terminal and C-terminal portions of hRluc together and restore its activity. The constructs were transfected into HEK293T cells in various combinations and hRluc activities were measured. As shown in Figure 1B, co-transfection of empty vectors (RN and RC) or Cby- and GFP-hRluc fusion plasmids (Cby-RN and GFP-RC or GFP-RN and Cby-RC) produced only a basal level of hRluc activity. In contrast, robust hRluc activity was observed when Cby-RN and Cby-RC were coexpressed. We also utilized a cell-permeable substrate for Renilla luciferase (ViviRen) to detect protein-protein interactions in live cells in real time. Figure 1C demonstrates that transient co-transfection of Cby-RN and Cby-RC expression vectors into HEK293T cells resulted in a high luminescence value, whereas either vector in combination with an empty plasmid (RC or RN) generated only low background luminescence. Interactions between Cby and the well-established basic leucine zipper (bZIP) coiled-coil transcription factors, Jun and Fos, were also tested using the ViviRen live cell substrate. No marked luminescence was detected when Cby-RN and Fos-RC or Jun-RN and Cby-RC were coexpressed in HEK293T cells, compared to that of Jun-Fos and Cby-Cby interactions, suggesting that the Cby-Cby interaction is specific.

Furthermore, we examined the stability of the Cby oligomer (Figure 1D). Flag-Cby and HA-Cby expression plasmids were co-transfected into HEK293T cells, and cell lysates were immunoprecipitated with anti-HA antibody. The immunoprecipitates were then exposed to increasing concentrations of NaCl or urea as indicated, resolved by SDS-PAGE, and analyzed by immunoblotting using anti-Flag antibody. Essentially, a similar amount of Flag-Cby was detected in the presence of up to 2 M NaCl or 2 M urea. These results indicate that the Cby oligomer is highly stable and that both electrostatic and hydrophobic interactions may contribute to the stability of the Cby complex.

### Cby is present as a homodimer

**Figure 2:**
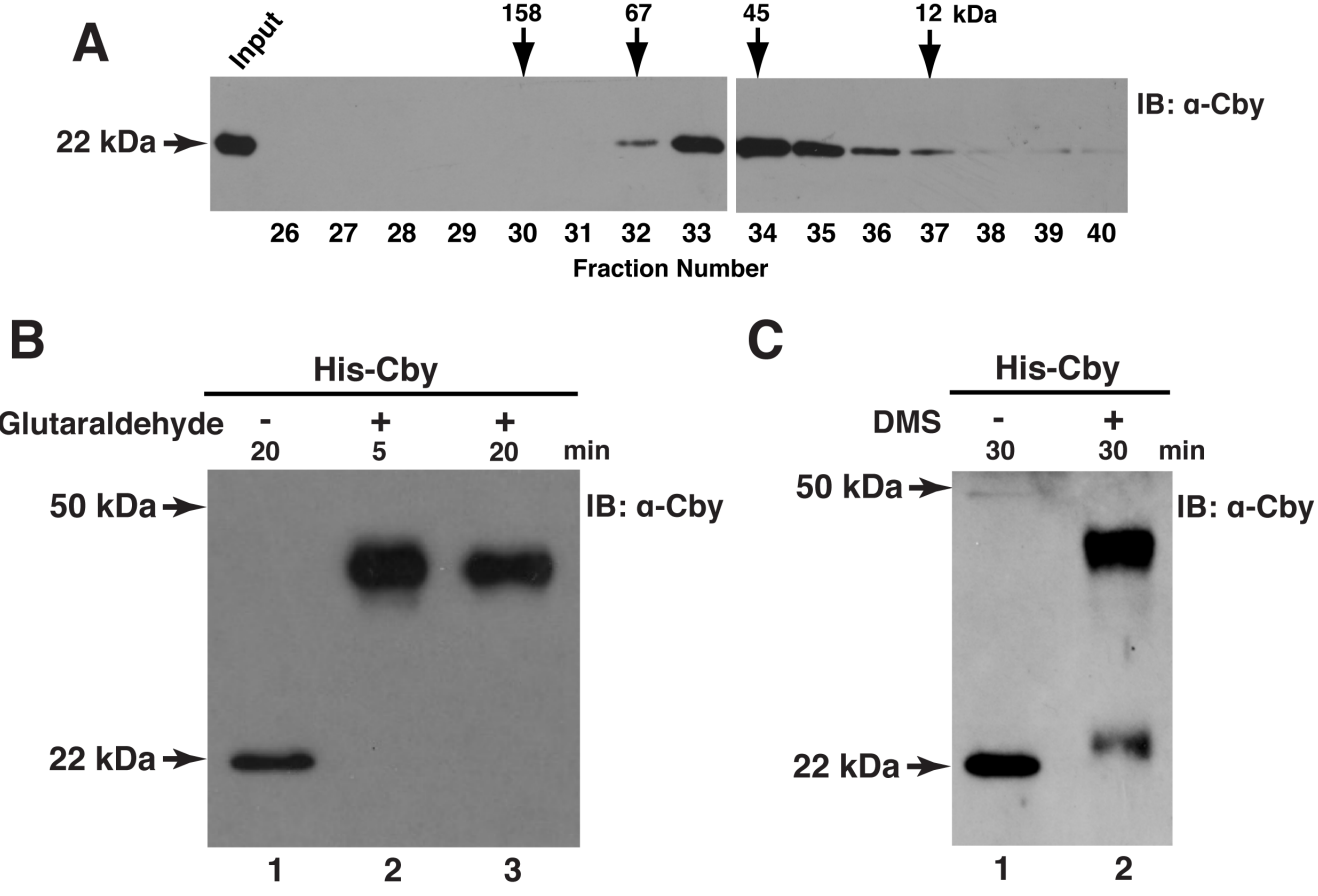
**Cby exists as a homodimer**. **(A) **Gel filtration analysis of Cby. His-Cby purified from stable HEK293T cells was run on a pre-calibrated Superdex 75 FPLC column in buffer containing 1 M NaCl. An aliquot of each fraction was resolved by SDS-PAGE and analyzed by Western blotting using anti-Cby antibody. The arrows indicate the elution positions of protein standards: aldolase, 158 kDa; bovine serum albumin, 67 kDa; ovalbumin, 45 kDa and cytochrome C, 12 kDa. Similar results were obtained using buffer containing 0.5 M NaCl (data not shown). **(B, C) **Cross-linking experiments. His-tagged Cby was purified from transiently transfected HEK293T cells using Ni-NTA beads, and subjected to cross-linking with glutaraldehyde for 5 or 20 min **(B)**, or dimethyl suberimidate (DMS) for 30 min **(C)**. The samples were then resolved by SDS-PAGE, followed by immunoblotting with anti-Cby antibody.

To gain insights into the oligomeric nature of Cby, we performed gel filtration chromatography. His-tagged Cby was purified from stable HEK293T cells using nickel beads and applied to a Superdex 75 column. Following fractionation, each fraction was examined by immunoblotting using anti-Cby antibody. As shown in Figure 2A, Cby eluted as a broad single peak centered at fraction 34, corresponding to a molecular weight of about 45 kDa. Since the molecular weight of a His-Cby monomer is 22 kDa, we estimated that Cby is most likely to be present as a homodimer.

As an independent means, we conducted glutaraldehyde cross-linking analysis. For this purpose, His-tagged Cby was transiently expressed in HEK293T cells, and purified using nickel beads. The purified Cby protein was then incubated in the presence or absence of glutaraldehyde for 5 or 20 min, and the cross-linked proteins were resolved by SDS-PAGE, followed by Western blot analysis with anti-Cby antibody (Figure 2B). As expected, in the absence of glutaraldehyde, His-Cby had an apparent monomer molecular weight of 22 kDa. On the other hand, after incubating with glutaraldehyde, His-Cby migrated slower at a higher molecular weight of approximately 44 kDa. Similar results were also observed using dimethyl suberimidate (DMS), another cross-linking reagent that contains a longer spacer arm (Figure 2C). All together, these results suggest that Cby predominantly exists as a homodimer.

### Cby harbors a putative leucine zipper coiled-coil motif in its C-terminal region

**Figure 3:**
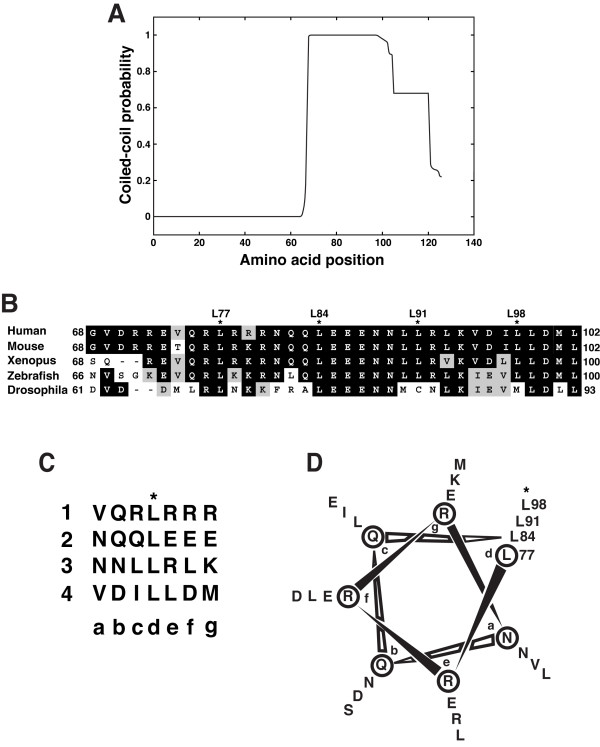
**The C-terminal region of Cby harbors a putative coiled-coil motif**. **(A) **The human Cby protein sequence (126 amino acid residues) was analyzed by the COILS program at a window size of 21 residues. Note that high coiled-coil probabilities exceeding 0.9 were evident from amino acid 68 to 102. **(B) **Sequence alignment of the Cby coiled-coil domain across species. Identical and similar residues are highlighted in black and gray, respectively. Four leucines, indicated by asterisks, appear at every seventh position, indicative of a leucine-zipper motif. **(C) **The putative coiled-coil motif of human Cby is depicted as heptad repeats of seven amino acids. The letters a through g designate the positions of residues within the heptad with hydrophobic residues generally found at the a and d positions. The four consecutive heptads are numbered 1 through 4. Note that the d positions are occupied by leucine residues.**(D) **Helical wheel diagram of the coiled-coil domain of human Cby (amino acids 77–98).

In our previous study [19], through inspection of the Cby protein sequence, we noted the presence of a putative coiled-coil motif in its C-terminal region. Coiled coils are a supercoiled bundle of α-helices known to mediate protein oligomerization [29,30]. Further analysis of the human Cby amino acid sequence using the COILS program [31,32] predicted a high probability of an α-helical coiled-coil structure extending from amino acids 68 to 102 (Figure 3A). Visual examination of this segment revealed four leucine residues that appear at every seventh position (Figure 3B), characteristic of a leucine zipper coiled-coil motif [33,34]. The sequence alignment of the Cby coiled-coil domain across species shows that these four leucines are perfectly conserved in vertebrate Cby homologues. On the contrary, only the first two leucine residues (L77 and L84) are conserved in fruit fly *D. melanogaster *Cby. Nonetheless, the corresponding region of *D. melanogaster *Cby was also predicted to form a coiled-coil structure by the COILS program (data not shown), signifying the functional importance of this domain. As depicted in Figure 3C, the coiled-coil domain of human Cby comprises four consecutive heptad repeats with amino acid positions labeled as a-g. Helical wheel projection of this region illustrates that the d positions are lined with leucine residues (Figure 3D), as typically observed in bZIP transcription factors [33,34]. The a and d positions are occupied generally by hydrophobic residues that interact with each other to form a hydrophobic core, while residues in the other positions are often surface-exposed [29,33,34]. Some of these exposed amino acid residues in the heptad repeats of Cby might directly contact β-catenin as the β-catenin-binding domain of Cby resides in its C-terminal half [19]. Collectively, these observations raise the possibility that the four heptad leucines may play an essential role in mediating Cby homodimerization.

### Cby self-interaction is mediated by the conserved leucine residues within the C-terminal heptad-repeat motif

**Figure 4:**
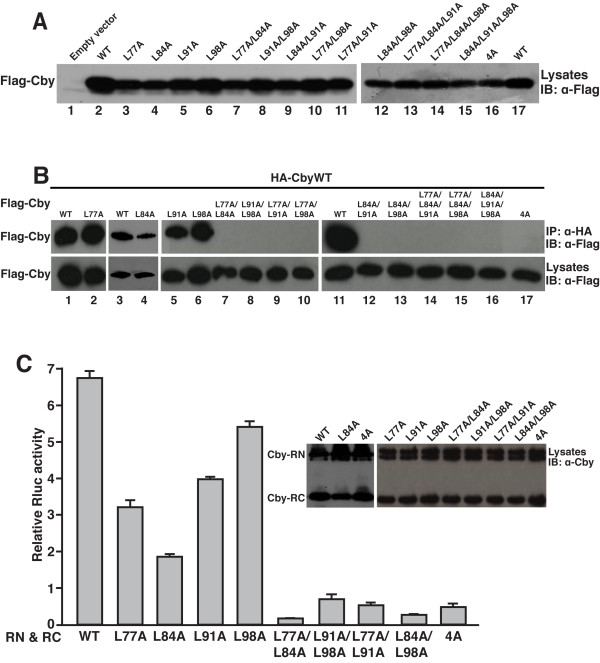
**The heptad leucine residues within the coiled-coil domain are crucial for Cby homodimerization**. **(A) **Expression levels of Cby point mutants. Lysates from HEK293T cells transfected with an equal amount (600 ng) of an expression plasmid for Flag-tagged wild-type (WT) or mutant Cby were subjected to Western blotting with an anti-Flag antibody. **(B) **Cell lysates were prepared from HEK293T cells transiently co-transfected with HA-CbyWT and Flag-CbyWT or individual Cby variants with all possible combinations of leucine-to-alanine mutations [single, double, triple and quadruple (4A)], and subjected to immunoprecipitation with anti-HA antibody. The immunoprecipitates were separated by SDS-PAGE and immunoblotted with anti-Flag antibody. To ensure sufficient protein expression levels, the amounts of plasmid DNA expressing Flag-tagged Cby mutants were increased by 2-fold for transfection, compared to those of Flag-CbyWT plasmid. **(C) **Split hRluc protein-fragment-assisted complementation assays using Cby point mutants. CbyWT or each of the indicated Cby mutants was fused in-frame to RN and RC. These expression plasmids (400 ng each) were transfected into HEK293T cells, and Rluc activities were measured as described in the legend to Figure 1B. Transfections were carried out in triplicate and the means ± SD are shown. Immunoblotting with anti-Cby antibody showed that the fusion proteins were stably expressed. Cby-RN fusion proteins appear as a doublet probably due to degradation.

To test the hypothesis that the four leucine residues in the C-terminal coiled-coil motif (L77, L84, L91 and L98) are critical for Cby self-assembly, we performed site-directed mutagenesis to substitute each leucine for an alanine in all possible combinations. These mutations would not be expected to disrupt the α-helical structure of Cby, but the shorter alanine side chain would not be able to mediate a leucine zipper-mediated stable coiled-coil configuration. Upon overexpression of N-terminally Flag-tagged proteins in HEK293T cells, all Cby mutants were detected at appreciable levels by Western blotting using anti-Flag antibody (Figure 4A). Next, the Cby mutants were evaluated for their ability to form a complex with wild-type Cby (CbyWT) by coimmunoprecipitation assays (Figure 4B). Individual Flag-tagged Cby mutant was coexpressed with HA-tagged CbyWT in HEK293T cells, and cell lysates were immunoprecipitated with anti-HA antibody and subjected to Western blot analysis with anti-Flag antibody. Cby single-point mutants were all able to interact with CbyWT although reduced interactions were noted for CbyL84A. Remarkably, Cby mutants carrying alanine substitutions for two or more out of the four leucine residues completely abolished their interaction with CbyWT. To validate these data, we carried out split hRluc assays for self-associations between Cby point mutants (Figure 4C). Coexpression of CbyWT-hRluc fusion proteins in HEK293T cells yielded high Rluc activity. Consistent with the coimmunoprecipitation experiments, reduced, yet significant Rluc activities were observed for Cby single-point mutants, whereas all Cby double mutants or the quadruple mutant (Cby4A) displayed only marginal Rluc activities.

**Figure 5:**
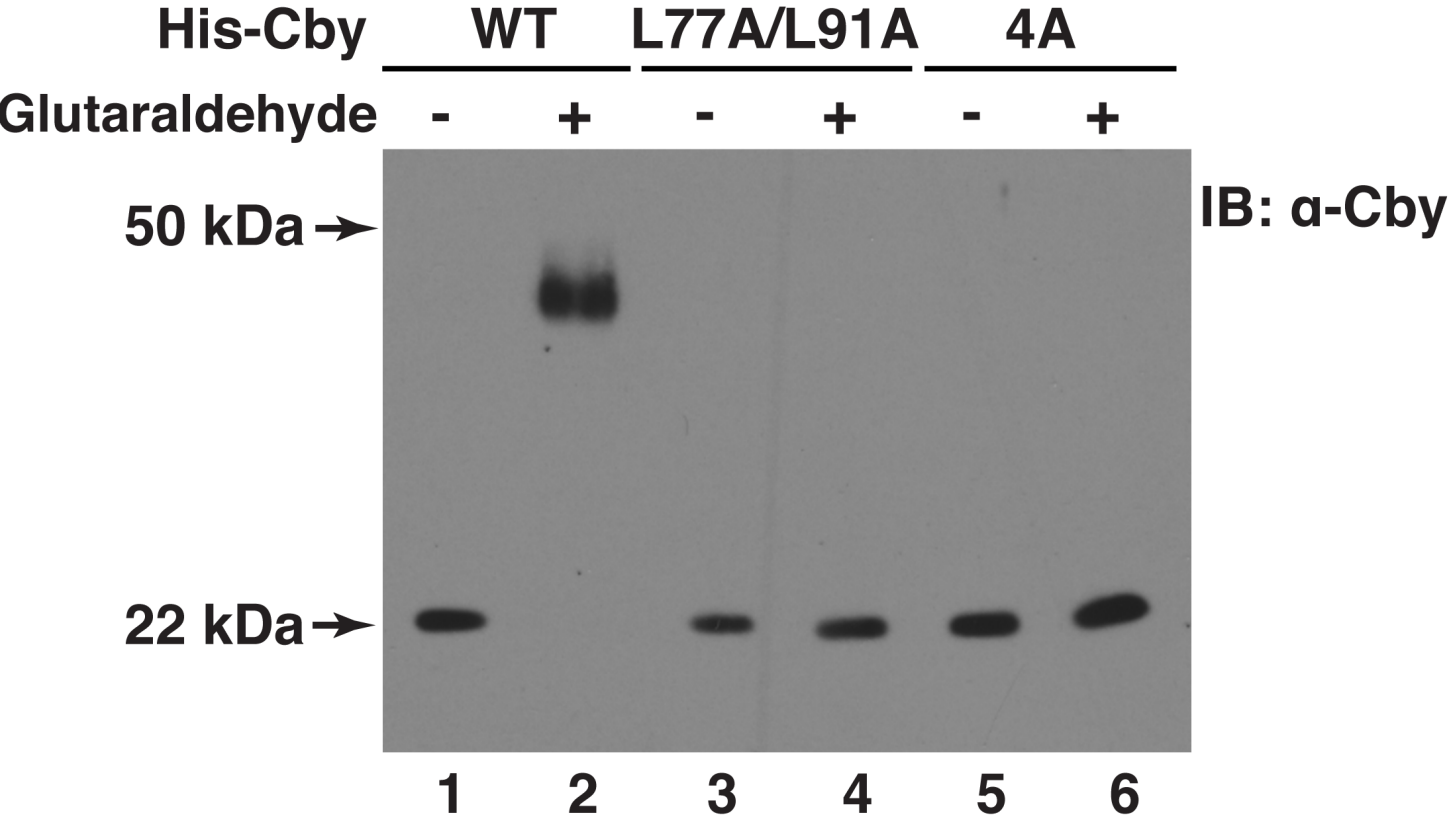
**Glutaraldehyde cross-linking of dimerization-deficient Cby mutants**. His-tagged CbyWT, L77A/L91A or 4A was transiently expressed in HEK293T cells, and purified using Ni-NTA beads. The purified Cby protein was incubated in the absence or presence of glutaraldehyde for 5 min, and resolved on SDS-PAGE, followed by immunoblotting with anti-Cby antibody.

To investigate if Cby mutants incapable of self-interaction are compromised in homodimer formation, we performed glutaraldehyde cross-linking experiments. As shown in Figure 5, CbyWT migrated as a dimer (lane 2) but CbyL77A/L91A and Cby4A migrated as a monomer (lanes 4 and 6, respectively) in SDS-PAGE. These data clearly highlight the functional importance of the heptad leucine residues in mediating Cby homodimerization.

### Cby self-association is not required for its interaction with β-catenin and inhibition of β-catenin-dependent transcriptional activation

**Figure 6:**
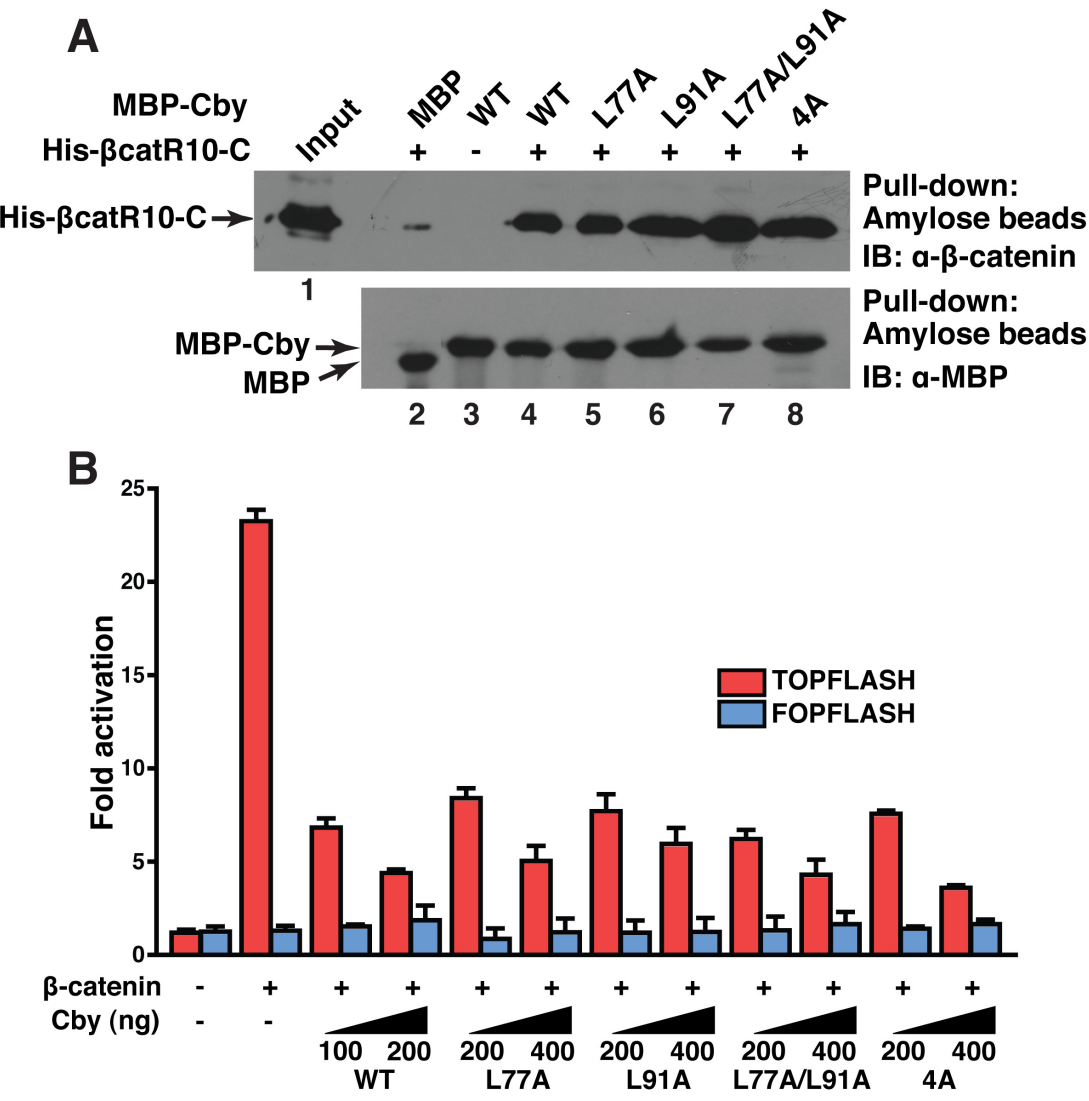
**Cby homodimerization is dispensable for its interaction with β-catenin and for repression of β-catenin signaling activity**. **(A) **Binding of Cby point mutants to β-catenin was evaluated by *in vitro *pull-down assays. Bacterially produced MBP or individual MBP-Cby protein was incubated with His-tagged β-catenin C-terminal domain (His-βcatR10-C). The protein complexes were then pulled down with amylose resin and subjected to Western blotting using anti-β-catenin antibody (top panel). The input lane was loaded with one-fiftieth of the amount of His-βcatR10-C used in the binding reactions (lane 1). One-thirtieth of each pull-down sample was run on a separate SDS-PAGE and immunoblotted with anti-MBP antibody, showing that similar amounts of MBP-Cby protein were pulled down (bottom panel). **(B) **The ability of Cby mutants to repress β-catenin signaling was tested by TOPFLASH assays. HEK293T cells were transfected with 60 ng of TOPFLASH or mutant FOPFLASH luciferase reporter, with or without 40 ng of an expression vector for stabilized β-catenin (β-catenin-Myc), and the indicated amounts of a Flag-tagged Cby expression vector. Luciferase activity was measured 24 hr post-transfection, and normalized to Renila luciferase activity used as an internal control. Transfections were carried out in triplicate and the means ± SD are shown. Note that, to compensate protein levels, higher amounts of plasmid DNA for the Cby mutants were used for transfection.

Cby physically interacts with the C-terminal activation domain of β-catenin, and inhibits β-catenin-dependent gene activation [19]. We took advantage of Cby dimerization-deficient mutants to examine whether the homodimer formation of Cby is a prerequisite for controlling β-catenin signaling. First, we assessed binding of Cby mutants to β-catenin using *in vitro *pull-down assays (Figure 6A). Bacterially expressed and purified maltose-binding protein (MBP) or MBP-CbyWT, L77A, L91A, L77A/L91A or 4A was incubated with His-tagged β-catenin C-terminal region (Armadillo repeat 10 to the C-terminus; β catR10-C) and pulled down with amylose beads. The bound proteins were then resolved by SDS-PAGE and analyzed by immunoblotting using anti-β-catenin antibody. Intriguingly, all the Cby point mutants tested, including the ones defective in homodimerization (CbyL77A/L91A and Cby4A), efficiently bound to β-catenin. Next, we performed Tcf/Lef luciferase reporter (TOPFLASH) assays [35] in HEK293T cells using the Cby mutants. Transfection of stabilized β-catenin activated TOPFLASH activity near 25-fold (Figure 6B). Co-transfection of CbyWT potently repressed β-catenin-mediated transcriptional activation in a dose-dependent manner. In agreement with the coimmunoprecipitation data, all the Cby point mutants inhibited TOPFLASH activation by β-catenin to an extent similar to that of CbyWT. In all cases, no or little changes were observed with the control reporter FOPFLASH carrying mutated Tcf/Lef-binding sites. Taken together, these results suggest that the monomeric form of Cby is sufficient for binding to β-catenin and for inhibiting β-catenin signaling activity.

### Cby homodimerization ensures its efficient nuclear entry

Our recent studies indicate that Cby harbors a functional nuclear localization signal (NLS) at the C-terminal end and a nuclear export signal (NES) in the N-terminal region, and constitutively shuttles between the nucleus and cytoplasm [25] (F.-Q. Li *et al*., manuscript submitted). Worthy of note, it has been reported that dimerization is a prerequisite process for the nuclear import of STAT, SREBP2 and viral IE1 transcription factors [36-38].

**Figure 7:**
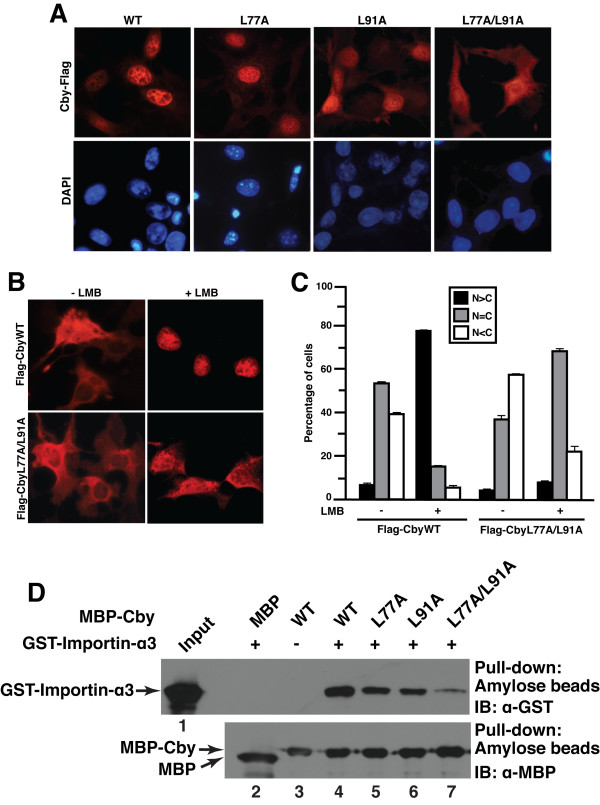
**Homodimer formation of Cby is a prerequisite for its efficient nuclear import**. **(A) **C-terminally Flag-tagged CbyL77A/L91A accumulates in the cytoplasm. COS7 cells were transiently transfected with an expression vector encoding C-terminally Flag-tagged WT or mutant Cby as indicated. Cells were fixed 24 hr post-transfection, followed by immunostaining with anti-Flag antibody. Nuclei were stained with DAPI. **(B) **A fraction of N-terminally Flag-tagged CbyL77A/L91A is found in the cytoplasm after LMB treatment. COS7 cells were transfected with an expression vector for N-terminally Flag-tagged CbyWT or CbyL77A/L91A, treated with methanol (- LMB) or 40 nM LMB (+ LMB) for 5 hr, and immunostained with anti-Flag antibody for Cby. Nuclei were counterstained with DAPI. **(C) **Quantitative analysis of the results in **(B)**. The subcellular localization of Flag-CbyWT and Flag-CbyL77A/L91A was scored as follows: N>C, predominantly nuclear; N = C, evenly distributed between the nucleus and cytoplasm; N<C, predominantly cytoplasmic. Error bars represent the means ± SD of three independent experiments. **(D) **Reduced binding of CbyL77A/L91A to importin-α. Bacterially produced MBP or the indicated MBP-Cby protein was incubated with GST-importin-α3. The complexes were then pulled down with amylose resin and subjected to Western blotting using anti-GST antibody (top panel). The input lane was loaded with one-fiftieth of the amount of GST-importin-α3 used in the binding reactions (lane 1). One-thirtieth of each pull-down sample was run on a separate SDS-PAGE and immunoblotted with anti-MBP antibody, showing that similar amounts of MBP-Cby protein were pulled down (bottom panel).

To begin to understand the biological significance of Cby homooligomerization, we assessed the subcellular localization of Cby mutants. In contrast to the diffuse cytoplasmic and nuclear distribution of N-terminally tagged CbyWT (Figure 7B) [25] and untagged CbyWT (data not shown), we noted that C-terminally tagged CbyWT (CbyWT-Flag) shows almost complete nuclear localization (Figure 7A), most likely due to a conformational change that exposes the normally buried C-terminal NLS. Notably, the dimerization-deficient mutant CbyL77A/L91A-Flag exhibited increased cytoplasmic staining, while both CbyL77A-Flag and CbyL91A-Flag mutants capable of self-interaction displayed predominant nuclear localization (Figure 7A). To confirm these results, we next evaluated the intracellular localization of N-terminally Flag-tagged Cby protein (Flag-Cby). Consistent with our previous observation [25], when expressed in COS7 cells, Flag-CbyWT primarily localized to the cytoplasm or both the nucleus and cytoplasm, especially in cells with high levels of Cby expression (Figure 7B and 7C). This cellular localization pattern of Flag-CbyWT is essentially the same as that of untagged protein (data not shown). In contrast, Flag-CbyL77A/L91A defective in dimer formation showed a subtle yet significant shift towards cytoplasmic localization. This trend became more apparent when cells were treated with leptomycin B (LMB), a potent inhibitor of the nuclear export receptor CRM1 [39,40]. Following LMB treatment, Flag-CbyWT was mostly nuclear but a significant fraction of Flag-CbyL77A/L91A was found in the cytoplasmic compartment (Figure 7B and 7C). A similar trend was observed with C-terminally Flag-tagged Cby proteins (Additional file 1). These findings suggest that homodimer formation of Cby is required for its efficient nuclear import.

Our recent data indicate that the C-terminal classical NLS of Cby physically interacts with the nuclear import receptor importin-α (F.-Q. Li *et al*., manuscript submitted). In the classical nuclear import pathway, importin-α plays a pivotal role by directly binding to NLS-containing proteins, linking them to importin-β [41-43]. Importin-β in turn docks the ternary import complex at the nuclear pore complex (NPC) and facilitates its translocation into the nucleus. In order to explore the molecular basis underlying the nuclear import defect of Cby homodimer mutants, we investigated interactions between Cby mutants and importin-α3 using *in vitro *binding assays. Bacterially produced MBP or MBP-Cby was incubated with GST-importin-α3 and pulled down using amylose resin. After extensive washing, the bound proteins were separated on SDS-PAGE and immunoblotted with anti-GST antibody. As shown in Figure 7D, the Cby single point mutants L77A and L91A bound to importin-α3 albeit with a slightly reduced affinity in comparison with CbyWT. On the other hand, the dimerization-defective CbyL77A/L91A exhibited a marked reduction in its ability to interact with importin-α3. Collectively, our results suggest that Cby homodimerization is a prerequisite for its efficient interaction with importin-α and subsequent nuclear translocation.

## Discussion

The canonical Wnt/β-catenin signaling pathway is essential for numerous cellular processes during embryonic development, tissue homeostasis and tumorigenesis. Upon Wnt stimulation, β-catenin, the key downstream component of this pathway, enters the nucleus and acts as a transcriptional coactivator to up-regulate target gene expression. Cby is an evolutionarily conserved protein that directly binds to the C-terminal activation domain of β-catenin and antagonizes its signaling activity [19]. Cby has been shown to interact with itself through its C-terminal region using yeast two-hybrid assays [27]. However, the molecular basis for the Cby-Cby interaction has remained largely uncharacterized to date.

Here we provide evidence that Cby forms a homodimer via the leucine zipper coiled-coil motif in its C-terminal region. The Cby complex is highly stable even in the presence of 2 M NaCl or 2 M urea (Figure 1D). We found that the four leucine residues in the d position of the helical wheel diagram (amino acids 77, 84, 91 and 98 in Figure 3D) are crucial for mediating Cby self-association. Alanine mutagenesis of two or more leucines completely abolishes the Cby-Cby interaction (Figures 4 and 5). Furthermore, our data indicate that the Cby-Cby interaction is not obligatory both for binding to β-catenin and for suppressing its signaling activity (Figure 6). Instead, homodimerization of Cby is a prerequisite process for its efficient binding to importin-α and nuclear import (Figure 7).

α-Helical coiled-coil motifs are versatile domains that mediate numerous protein-protein interactions [29,30]. The C-terminal coiled-coil motif of Cby is evolutionarily conserved from fly to human, implying the biological importance of this domain. Consistent with the presence of the α-helical coiled-coil structure, circular dichroism (CD) studies showed that Cby protein has a high α-helical content [44]. Our cross-linking and gel filtration experiments suggest that Cby predominantly forms a dimer (Figure 2). However, it is also possible that Cby exists in a larger oligomeric state under certain conditions as shown for other coiled-coil proteins [29,30]. In order to gain further insights into the molecular basis and functional significance of the Cby-Cby interaction, it would be of great interest to determine the three-dimensional structure of the Cby complex. However, so far, Cby protein is insoluble and forms inclusion bodies when expressed using bacterial as well as baculoviral expression systems (data not shown), thereby impeding further structural and biochemical analyses. We found that the aggregation of Cby protein is mainly caused by the C-terminal coiled-coil domain since the N-terminal half of the protein is highly soluble in *E. coli *(data not shown).

Our recent data suggest that Cby harbors functional NLS and NES motifs, and constantly cycles between the nucleus and cytoplasm. Thus, it appears that Cby intracellular localization at steady state is determined by a dynamic balance between its nuclear import and export. In this respect, Cby homodimerization represents a crucial step for its efficient binding to importin-α and subsequent nuclear import. It is noteworthy that dimer formation has been shown to be the key regulatory event controlling the nuclear entry of STAT, SREBP2 and viral IE1 transcription factors [36-38]. Nuclear import of STATs is mediated by importin-α family members [37,45], but that of SREBP2 depends on importin-β [38]. Interestingly, binding of importin-α5 to STAT dimers strictly requires two intact NLS elements, one in each STAT monomer [37]. We therefore envision that the assembly of Cby into dimers creates a fully functional NLS perhaps by juxtaposing the NLS of each monomer, allowing its efficient binding to importin-α and subsequent nuclear import. Alternatively, Cby oligomerization may induce a conformational change that unmasks the NLS.

Our results clearly show that Cby mutants deficient in self-interaction are capable of efficiently binding to β-catenin, leading to repression of its signaling activity to a similar extent as wild-type Cby (Figure 6), despite the fact that these Cby mutants are predominantly cytoplasmic (Figure 7). However, this is in agreement with our model in which Cby inhibits β-catenin signaling through two distinct molecular mechanisms [25,26]: 1) in the nucleus, Cby competes with Tcf/Lef transcription factors for binding to β-catenin; 2) Cby sequesters β-catenin in the cytoplasm in collaboration with 14-3-3 proteins. Thus, we speculate that monomeric Cby suppresses β-catenin signaling by trapping β-catenin within the cytoplasmic compartment. Alternatively, a small portion of nuclear Cby might be sufficient to repress β-catenin-mediated transcriptional activation since dimerization-deficient Cby mutants are able to enter the nucleus, albeit at a reduced rate (Figure 7B).

It is possible that the Cby-Cby interaction is of importance in certain biological contexts other than β-catenin signaling. For example, Cby has been shown to interact with thyroid cancer-1 (TC-1) [46] and polycystin-2 (PC-2) [27]. TC-1 was initially identified as a gene whose expression was elevated in thyroid cancers [47]. More recently, TC-1 was shown to interact with Cby and stimulate β-catenin signaling presumably by displacing Cby from β-catenin [46]. However, its precise functions are not yet completely understood. PC-2 is another known binding partner of Cby [27]. The PDK-2 gene, encoding PC-2, is mutated in patients with autosomal dominant polycystic kidney disease [48,49]. A previous report showed that Cby associates with PC-2 and regulates its subcellular distribution [27]. Hence, Cby appears to exert multiple biological functions. Whether Cby self-association is involved in controlling the activity of TC-1 and PC-2 awaits further investigation.

## Conclusion

In the present study, we demonstrated that Cby forms a stable dimer through its C-terminal coiled-coil motif, which consists of a heptad repeat of four conserved leucine residues. Our extensive mutational analysis clearly shows that these leucines are critical for Cby dimerization. In addition, our results indicate that the Cby self-interaction is dispensable for inhibition of β-catenin signaling but is required for Cby nuclear import.

## Methods

### Plasmids

Expression vectors for Flag-CbyWT, MBP-CbyWT, β-catenin-Myc and His-β catR10-C have been previously described [19,50]. The GST-importin-α3 construct was a kind gift from Dr. Nancy Reich at SUNY at Stony Brook [45]. To generate Myc- and HA-tagged CbyWT expression constructs, human Cby cDNA was excised from the Flag-CbyWT vector with Eco RI and Xho I, and subcloned into pCS2+Myc and pCS2+HA, respectively. A C-terminally Flag-tagged CbyWT (CbyWT-Flag) plasmid was generated by PCR amplification of human Cby cDNA using a 3' primer containing a Flag sequence, digested with Eco RI and Xho I and subcloned into pCS2+. Both N- and C-terminally Flag-tagged Cby point mutants were created with the QuickChange site-directed mutagenesis kit (Stratagene) using the Flag-tagged CbyWT vectors as templates. To obtain His-CbyWT mammalian expression plasmid, Cby cDNA was PCR-amplified, digested with Eco RI and Xho I, and subcloned into pcDNA4/HisMax A (Invitrogen). His-CbyL77A/L91A and His-Cby4A expression vectors were constructed by digesting the His-CbyWT construct with Bam HI and Xho I, and replacing its insert with the corresponding Cby mutant fragment from Flag-CbyL77A/L91A and Flag-Cby4A plasmids, respectively. To construct MBP-Cby mutants, the cDNA inserts were PCR-amplified, digested with Bgl II and Xho I, and ligated into pMAL-c2 (New England Biolabs). For synthetic Renilla luciferase (hRluc) protein-fragment-assisted complementation assays [28], cDNAs encoding Cby, GFP, Jun or Fos were amplified by PCR using plasmid templates, and ligated in-frame with the N-terminal portion (amino acids 1–239) or the C-terminal portion (amino acids 240–321) of hRluc into the pJCH510 or pJCH511 vector [25]. All constructs were verified by DNA sequencing.

### Cell culture and transfection

HEK293T cells were purchased from ATCC, and maintained in DMEM with 10% FBS and 100 units/ml penicillin-streptomycin. For transient transfection, cells were seeded onto 6- or 12-well tissue culture dishes, cultured overnight, and then transfected using Lipofectamine 2000 (Invitrogen) or SuperFect (Qiagen) according to the manufacturer's instructions. Empty vector was added to adjust the total amount of DNA to be the same in every transfection. To establish stable HEK293T cells, the His-CbyWT expression plasmid was transfected into HEK293T cells and selected with 500 μg/ml Zeocin (Invitrogen).

### Coimmunoprecipitation and Western blotting

HEK293T cell lysates were prepared in lysis buffer containing 20 mM Tris-HCl, pH 8.0, 135 mM NaCl, 1.5 mM MgCl_2_, 1 mM EGTA, 1% Triton X-100, 10% glycerol and complete protease inhibitor cocktail (Roche), and cleared by centrifugation at 12,000 rpm for 30 min at 4°C. Coimmunoprecipitation and immunoblotting were performed as previously described [23,25], except that increasing concentrations of NaCl and urea were included in wash buffer for the coimmunoprecipitation experiments shown in Figure 1D. The primary antibodies used were: mouse anti-Flag M2 (Sigma); mouse anti-Myc 9E10 (Invitrogen); rat anti-HA (Roche); rabbit anti-Cby [19]; rabbit anti-β-catenin (Sigma); rabbit anti-MBP (New England Biolabs); mouse anti-GST (Novagen).

### Synthetic Renilla luciferase (hRluc) protein-fragment-assisted complementation and TOPFLASH assays

HEK293T cells were seeded onto 12-well plates and transfected with appropriate combinations of plasmids. Luciferase activities were measured using the Dual Luciferase Reporter Assay System (Promega) and a Berthold luminometer as previously described [23,25]. An expression plasmid (10 ng) for Renilla luciferase (pRL-TK) or (5 ng) of firefly luciferase (pCMV-Luc) was co-transfected to normalize transfection efficiency. For the split hRluc assays using ViviRen live cell substrate in Figure 1C, cells were seeded onto 96-well plates and transfected with appropriate combinations of hRluc fusion constructs. The next day, ViviRen (Promega) was directly added to the tissue culture media and Renilla luciferase luminescence was measured according to the manufacturer's instructions.

### Cross-linking experiments and gel filtration chromatography

His-tagged CbyWT, L77A/L91A and 4A were transiently expressed in HEK293T cells, and purified using Ni-NTA His-Bind Resin (Novagen) according to the manufacturer's instructions. The purified proteins were dialyzed against dialysis buffer containing 20 mM Hepes, pH 7.9, 100 mM NaCl, 1 mM EDTA, 0.1% NP-40 and 10% glycerol. An aliquot of the protein samples was incubated in the absence or presence of freshly prepared glutaraldehyde (0.2% final concentration) (Sigma) at 37°C for 5 or 20 min or dimethyl suberimidate (DMS; 2 mg/ml final concentration) (Sigma) at room temperature for 30 min in a 40 μl of the dialysis buffer. The glutaraldehyde reaction was stopped by addition of 10 μl of 1 M Tris-HCl, pH 8.0. The samples were then mixed with SDS sample buffer, boiled and resolved by SDS-PAGE, followed by immunoblotting with anti-Cby antibody.

For the gel filtration experiment, His-Cby was purified from ten 15-cm dishes of stable HEK293T cells, and dialyzed against the dialysis buffer as described above for cross-linking. The protein sample was then loaded onto a Superdex 75 gel filtration column (Amersham Biosciences), and run via fast protein liquid chromatography (FPLC) (Amersham Biosciences) with gel filtration buffer containing 20 mM Tris-HCl, pH 8.0, 0.5 or 1.0 M NaCl, 1 mM EDTA, 2 mM DTT, 2 mM betaine-HCl, 0.02% Triton X-100 and 5% glycerol at a flow rate of 0.3 ml/min. Fractions of approximately 0.5 ml were collected and analyzed by Western blotting with anti-Cby antibody. The column was calibrated using protein standards (Amersham Biosciences): ferritin, 450 kDa; aldolase,158 kDa; bovine serum albumin, 67 kDa; ovalbumin, 45 kDa; cytochrome C, 12 kDa.

### Protein expression in bacteria and in vitro pull-down assays

GST and MBP fusion and His-tagged proteins were expressed according to the manufacturer's instructions, and *in vitro *binding assays were performed essentially as described previously [19,50].

### Immunofluorescence microscopy

Transfected COS7 cells were grown on glass coverslips, fixed with methanol-acetone (1:1, v/v), permeabilized with 0.2% Triton X-100 and blocked with 1% BSA in PBS. Flag-tagged Cby was detected using mouse anti-Flag M2 antibodies (Sigma), followed by TRITC-labelled goat anti-mouse IgG (Jackson Immunoresearch Laboratories). Nuclei were counterstained with DAPI (Sigma) and stained cells were analyzed by a Leica DM5000 fluorescent microscope. To quantify subcellular localization, independent transfections were performed at least three times, and a minimum of 100 cells were counted for each transfection.

## Authors' contributions

AM, FQL and KIT conceived and designed the experiments; AM performed most of the experiments. FQL generated the Flag-Cby mutant expression plasmids; JCH constructed the pJCH510 and pJCH511 vectors and carried out the hRluc protein-fragment-assisted complementation assays using ViviRen live cell substrate; FQL and KIT supervised the study; AM and KIT drafted the manuscript; All authors have read and approved the final version of the manuscript.

## Supplementary Material

Additional file 1 **Cytoplasmic localization of the dimerization-defective mutant CbyL77A/L91A with a C-terminal Flag tag**. **(A) **COS7 cells were transiently transfected with an expression vector for C-terminally Flag-tagged CbyWT or CbyL77A/L91A, treated with methanol (- LMB) or 40 nM LMB (+ LMB) for 5 hr, and immunostained with anti-Flag antibody for Cby. Nuclei were counterstained with DAPI. Note that the majority of cells expressing CbyL77A/L91A-Flag showed cytoplasmic staining, whereas greater than 95% of cells expressing CbyWT-Flag showed almost exclusive nuclear localization. **(B) **Quantitative analysis of the results in **(A)**. The subcellular localization of Cby-Flag and CbyL77A/L91A-Flag was scored as follows: N>C, predominantly nuclear; N = C, evenly distributed between the nucleus and cytoplasm; N<C, predominantly cytoplasmic. Error bars represent the means ± SD of three independent experiments. Graphs displaying C-terminally Flag-tagged Cby proteins. Click here for file
