# Supplementary figures and images for: Chibby forms a homodimer through a heptad repeat of leucine residues in its C-terminal coiled-coil motif
Source: BMC Mol Biol. 2009 May 12;10:41. doi: 10.1186/1471-2199-10-41 (PMC2686680; doi:10.1186/1471-2199-10-41)

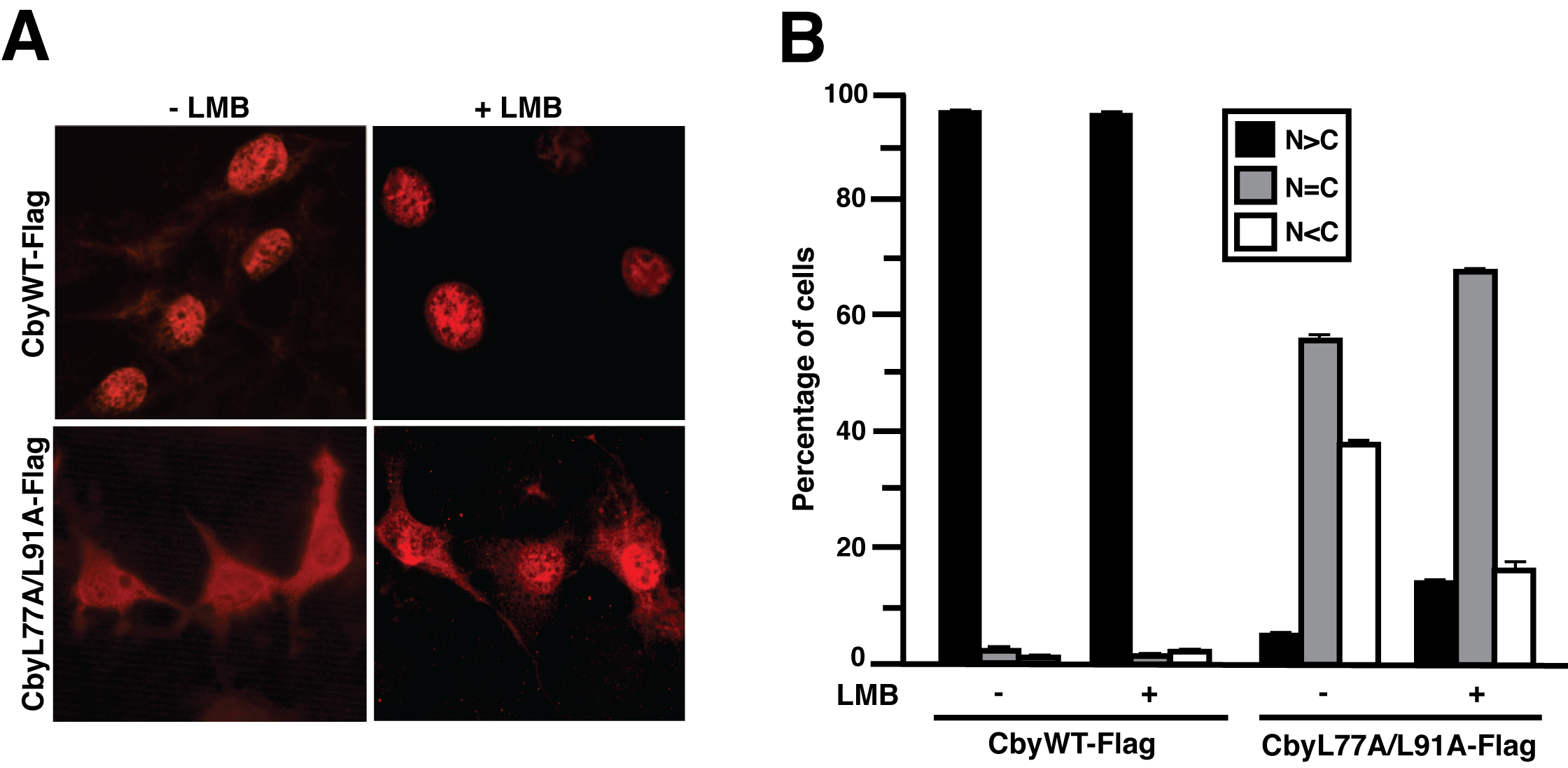

Supplement: Additional file 1 — Cytoplasmic localization of the dimerization-defective mutant CbyL77A/L91A with a C-terminal Flag tag. (A) COS7 cells were transiently transfected with an expression vector for C-terminally Flag-tagged CbyWT or CbyL77A/L91A, treated with methanol (- LMB) or 40 nM LMB (+ LMB) for 5 hr, and immunostained with anti-Flag antibody for Cby. Nuclei were counterstained with DAPI. Note that the majority of cells expressing CbyL77A/L91A-Flag showed cytoplasmic staining, whereas greater than 95% of cells expressing CbyWT-Flag showed almost exclusive nuclear localization. (B) Quantitative analysis of the results in (A). The subcellular localization of Cby-Flag and CbyL77A/L91A-Flag was scored as follows: N>C, predominantly nuclear; N = C, evenly distributed between the nucleus and cytoplasm; N<C, predominantly cytoplasmic. Error bars represent the means ± SD of three independent experiments. Graphs displaying C-terminally Flag-tagged Cby proteins. [file 1471-2199-10-41-S1.png]
